# Regulatory Elements Associated with Paternally-Expressed Genes in the Imprinted Murine Angelman/Prader-Willi Syndrome Domain

**DOI:** 10.1371/journal.pone.0052390

**Published:** 2013-02-04

**Authors:** Sara Rodriguez-Jato, Jixiu Shan, Jyoti Khadake, Arnold D. Heggestad, Xiaojie Ma, Karen A. Johnstone, James L. Resnick, Thomas P. Yang

**Affiliations:** 1 Department of Biochemistry and Molecular Biology, University of Florida College of Medicine, University of Florida, Gainesville, Florida, United States of America; 2 Department of Molecular Genetics and Microbiology, University of Florida College of Medicine, University of Florida, Gainesville, Florida, United States of America; 3 Center for Epigenetics, University of Florida College of Medicine, University of Florida, Gainesville, Florida, United States of America; Universität des Saarlandes, Germany

## Abstract

The Angelman/Prader-Willi syndrome (AS/PWS) domain contains at least 8 imprinted genes regulated by a bipartite imprinting center (IC) associated with the *SNRPN* gene. One component of the IC, the PWS-IC, governs the paternal epigenotype and expression of paternal genes. The mechanisms by which imprinting and expression of paternal genes within the AS/PWS domain – such as *MKRN3* and *NDN* – are regulated by the PWS-IC are unclear. The syntenic region in the mouse is organized and imprinted similarly to the human domain with the murine PWS-IC defined by a 6 kb interval within the *Snrpn* locus that includes the promoter. To identify regulatory elements that may mediate PWS-IC function, we mapped the location and allele-specificity of DNase I hypersensitive (DH) sites within the PWS-IC in brain cells, then identified transcription factor binding sites within a subset of these DH sites. Six major paternal-specific DH sites were detected in the *Snrpn* gene, five of which map within the 6 kb PWS-IC. We postulate these five DH sites represent functional components of the murine PWS-IC. Analysis of transcription factor binding within multiple DH sites detected nuclear respiratory factors (NRF's) and YY1 specifically on the paternal allele. NRF's and YY1 were also detected in the paternal promoter region of the murine *Mrkn3* and *Ndn* genes. These results suggest that NRF's and YY1 may facilitate PWS-IC function and coordinately regulate expression of paternal genes. The presence of NRF's also suggests a link between transcriptional regulation within the AS/PWS domain and regulation of respiration. 3C analyses indicated *Mkrn3* lies in close proximity to the PWS-IC on the paternal chromosome, evidence that the PWS-IC functions by allele-specific interaction with its distal target genes. This could occur by allele-specific co-localization of the PWS-IC and its target genes to transcription factories containing NRF's and YY1.

## Introduction

Angelman syndrome (AS) and Prader-Willi syndrome (PWS) are two distinct neurogenetic diseases associated with the same ∼2 Mb domain in human chromosomal region15q11–13 (reviewed in [1]). This region contains a domain of imprinted genes in which loss of expression of the paternally-inherited allele of genes including *SNURF-SNRPN*, *NECDIN* (*NDN*), *MAGEL2*, and *MKRN3* is associated with PWS. In contrast, AS is associated with loss of expression or mutation of the maternally expressed *UBE3A* gene [2]. The parent-of-origin epigenotype and expression of genes throughout the AS/PWS domain is coordinated by a bipartite imprinting center (IC) defined by microdeletions found in a subset of PWS and AS patients [3], [4], [5]. Mapping of microdeletions associated with PWS has identified a 4.3 kb region of the *SNURF-SNRPN* gene (hereafter, referred to as *SNRPN*) that appears to be required for establishment and/or maintenance of the paternal epigenotype across the imprinted AS/PWS domain [4], [6]. This region, referred to as the PWS-SRO (smallest region of deletion overlap), includes the promoter, 5′ flanking region, and 5′ portion of the first intron of *SNRPN*. Mapping of microdeletions associated with AS has identified a 0.88 kb region, termed the AS-SRO, that is located ∼ 35 kb upstream of exon 1 of *SNRPN* and appears to govern the maternal epigenotype [6], [7], [8], [9]. Thus, the bipartite IC consists of a PWS-IC and AS-IC that encompass the PWS-SRO and AS-SRO, respectively, and regulates establishment and/or maintenance of imprinting across the domain. Though the molecular mechanisms of IC function are not well-understood, the PWS-IC has been postulated to function as a positive regulator of genes expressed specifically from the paternally-inherited chromosome, while the AS-IC has been postulated to act as a negative regulator of the PWS-IC [9], [10]. A recent report also presents evidence of a role for the PWS-IC on the maternal chromosome [11]. Allele-specific expression of individual genes in the AS/PWS region has been shown to be correlated with differential DNA methylation, histone modification patterns, DNase I hypersensitivity, and transcription factor binding [1], [12], [13], [14], [15], [16], [17].

The region syntenic to the human AS/PWS domain on mouse chromosome 7 contains the orthologous genes organized in the same order with the same imprinted expression patterns as the human domain [1]. The minimal murine PWS-IC was originally defined by a targeted 35 kb deletion of the *Snrpn* locus [18], but recently has been further delineated by a targeted 6 kb deletion of the *Snrpn* gene that includes the 5′ flanking region, promoter, and 5′ portion of the 1st intron [19]. Both deletions resulted in a complete PWS-IC imprinting defect (e.g., loss of paternal gene expression, and maternal-specific DNA methylation patterns on paternal alleles) and neonatal lethality when paternally-inherited. Similarly, a deletion of the entire AS/PWS region in the mouse also showed a failure to thrive and postnatal lethality when the deletion was paternally transmitted [20]. The murine AS-IC has not been clearly delineated, though AS-IC activity has recently been attributed to several upstream promoters that drive transcripts through the PWS-IC in oocytes [21]. How this transcription may lead to epigenetic inactivation of the PWS-IC is unknown.

The imprinted mouse *Mkrn3* gene is a ubiquitously expressed, retrotransposed, intronless gene that encodes a RING zinc-finger protein of the Makorin family with unknown function [22], [23], [24]. It is located ∼2.4 Mb upstream of the murine PWS-IC and expressed exclusively from the paternally-inherited chromosome. Little is known about the mechanisms by which distal genes in the AS/PWS domain such as *Mkrn3* are regulated by the IC over large genomic distances to establish and/or maintain the correct epigenotype and imprinted patterns of gene expression.

To identify elements that may mediate PWS-IC function and potentially regulate imprinted expression of *Mkrn3* by the PWS-IC, we now have analyzed regulatory regions within the mouse *Snrpn*/PWS-IC and *Mkrn3* loci for transcription factor binding. We find recurring association of nuclear respiratory factors (NRF's) and YY1 within multiple regulatory regions exclusively on the paternally-inherited chromosome, suggesting a role for these factors in the coordinate parent-of-origin regulation of genes within this domain. The ubiquity of nuclear respiratory factors in this region also suggests a possible functional connection between regulation of genes in the AS/PWS domain and respiration. We also present evidence that the murine *Mkrn3* locus lies in close proximity to the PWS-IC specifically on the paternal chromosome and discuss the implications of this allele-specific spatial organization of the AS/PWS domain in the context of transcription factor binding patterns.

## Materials and Methods

### Mice and cell lines

Allele-specific analysis of the *Snrpn* gene (and PWS-IC) employed mice carrying a 35 kb PWS-IC deletion on either the maternally- or paternally-inherited chromosome [8], [18]. Allele-specific analyses were also facilitated by use of Tg^PWS(del)^ and Tg^AS(del)^ mice carrying a transgene-associated deletion of the entire AS/PWS region on either the paternally- or maternally-inherited chromosome, respectively, as well as immortalized fibroblast cell lines generated from these mice [20]. These mice and cells were generously provided by Robert Nicholls. Female Tg^AS(del)^ mice were bred with C57BL6/J male mice to obtain Tg^AS(del)^ mice for analysis; male Tg^AS(del)^ mice were bred with C57BL6/J females to obtain Tg^PWS(del)^ mice. Both cell lines were grown at 37°C in DMEM medium supplemented with 10% FBS and penicillin/streptomycin in 5% CO_2_. Experiments using primary mouse tissues were carried out in strict accordance with and approved by the University of Florida Institutional Animal Care and Use Committee (protocol number D586). Animals were humanely euthanized according to the *American Veterinary Medical Association Guidelines for Euthanasia* and all efforts were made to minimize suffering.

### Preparation of cell suspensions from mouse brain and spleen tissues

Primary mouse brain cell suspensions were prepared from 1–2 day newborn mice. Pups were humanely sacrificed by rapid decapitation and brain tissue was dissected and placed in ice cold phosphate buffered saline (PBS) containing 0.5 M sucrose. Dissected brains were transferred to fresh ice cold PBS/sucrose solution where blood vessels and other fibrous tissue were removed with forceps. Remaining brain tissue was then minced into ∼1–2 mm pieces using scissors and triturated gently in PBS/sucrose with a Pasteur pipette. At regular intervals, the triturated cell suspension was monitored by light microscopy to determine when the tissue was optimally dispersed into single cells (but not lysed to nuclei). The resulting cell suspension was filtered though a cheese cloth into a conical tube on ice. The remaining tissue fragments were further triturated in fresh PBS/sucrose, monitored for tissue dispersion to single cells, and the supernatant filtered and combined with the initial supernatant; cells in the pooled supernatants were used for the analyses described below. Single cell suspensions from primary mouse spleen cells were generated from dissected adult spleens by cutting spleens into sections and gently squeezing the tissue to release a suspension of single cells into a cell culture dish containing PBS and 0.5 M sucrose.

### DNase I hypersensitivity analysis

Analysis of DNase I hypersensitivity by Southern blot analysis was performed as described previously [17], [25]. For analysis of primary mouse brain cells, cell suspensions were prepared from one day old newborn mice as described above, permeablized cells were treated with increasing concentrations of DNase I (Worthington) in suspension, then purified genomic DNA from each sample was digested with Eco RI, Taq I, or Nco I, and Southern blotted. Hybridization probes for Southern blot analyses were isolated and radiolabeled by random priming as described previously [17], [25]. Hybridization probe A was a 475 bp fragment spanning positions +1397 to +1872 (relative to the transcription initiation site) of the *Snrpn* gene synthesized by PCR and cloned. Probe B was a 115 bp bp Pst I (+2214 from transcription initiation site)–EcoRI fragment (+2329) from plasmid pGN72 containing a BamHI-EcoR I fragment of the *Snrpn* gene (generously provided by Robert Nicholls; [26]). Probe C was a 248 bp fragment spanning positions +2120 to +2367 generated by PCR and cloned. For analysis of the *Mkrn3* locus, approximately 1×10^7^ Tg^PWSdel^ or Tg^ASdel^ cells were permeablized and treated with increasing concentrations of DNase I (Worthington), then purified DNA from DNase-treated samples was digested with EcoRI and Southern blotted. Blots were hybridized with a α-^32^P-dCTP radiolabeled 0.7 kb Bgl II-EcoRI fragment from the *Mkrn3* locus.

### *In vivo* footprinting

*In vivo* footprinting by ligation-mediated PCR (LMPCR) was performed essentially as described previously [17], [27], [28] with modifications to optimize results for each gene analyzed. For analysis of *Snrpn* in primary mouse brain, aliquots of dispersed primary brain cells were treated with increasing concentrations of dimethyl sulfate (DMS) and genomic DNA was purified. For LMPCR, the primer extension reaction (using the first primer in each set shown below) was performed with *Vent* DNA polymerase (New England Biolabs) using a deoxynucleotide triphosphate mix that included 7-deaza-dGTP at a ratio of 7-deaza-dGTP/dGTP of 3∶1 as previously described [29]. First strand synthesis with Vent polymerase was performed as follows: Genomic DNA was denatured at 96°C for 10 min. in 15 ul followed by annealing of the first primer of each set (TY381, 644, 557) at 45°C for 30 min. in 40 mM NaCl, 10 mM Tris-HCl, pH 8.9. The extension reaction was carried out in 30 ul by addition of Vent polymerase (0.5 units per reaction in 40 mM NaCl, 10 mM Tris-HCl, pH 8.9, 5 mM MgSO_4_, and 0.25 mM of each deoxynucleotide triphosphate) and cycling of the reaction at 59°C for 1 min., 53°C for 1 min., 57°C for 1 min., 60°C for 1 min., 68°C for 1 min., and 76°C for 1 min. After ligation of the linker primer, the ligated template was then subjected to PCR amplification of the appropriate region using *Taq* DNA polymerase with the gene-specific 2^nd^ primer of each LMPCR primer set (TY382, 643, 558) and the LMPCR linker primer. PCR amplification with Taq polymerase (Invitrogen) was performed in 40 mM NaCl, 10 mM Tris-HCl, pH 8.9, 1.75 mM MgCl_2_, 0.25 mM of each dNTP (with a 3∶1 7–deaza-dGTP/dGTP mixture substituted for dGTP). Reactions were initiated by denaturation of each sample at 94°C for 3 min. Then each sample was subjected to 23 cycles of the following: denaturation at 94°C for 20 sec., annealing of the PCR primers for 1 min. at the temperature shown for each gene-specific 2^nd^ primer (TY382, 643, 558), extension at 76°C for 1 min. with an incremental increase of 3 sec. per cycle, and a final extension at 76°C for 10 min. after the PCR final cycle. Gel electrophoresis of the LMPCR-amplified DNA was carried out on 5% denaturing Long Ranger DNA sequencing gels, and electrotransfer of the gel onto Hybond N+ nylon membrane (Amersham) was performed in an electroblotting apparatus (at 110 V in a transfer buffer of 40 mM Tris, 40 mM boric acid, 1.6 mM EDTA, pH 8.0) as described previously [27]. Synthesis of radiolabeled probes from single-stranded templates and hybridization to electrotransferred membranes were performed as previously described [27], [30]. Briefly, a fragment of the *Snrpn* gene from positions −305 bp to +308 bp (relative to the transcription initiation site) was cloned into M13 mp18 and M13mp19 to obtain single-stranded clones M13mp1873 and M13mp1973 containing the sense and antisense strands, respectively. These single-stranded DNA templates were used to prepare appropriate strand-specific probes of interest by primer extension using the second LMPCR primer from the region of interest. The probe for analysis of the lower strand was synthesized from template M13mp1973, while probes for analysis of the upper strand were synthesized from M13mp1873. Probes were synthesized by primer extension using the Klenow fragment of DNA polymerase in the presence of 10 uCi of α-^32^P-dCTP. Purification of the radiolabeled probe was carried out in 6% acrylamide gels in TBE (100 mM Tris, 100 mM boric acid, 4 mM EDTA) by excising the probe from the gel, then macerating the gel fragment in hybridization buffer (0.25 M Na_2_HPO_4_ [pH 7.2], 7% SDS, 1% fraction V BSA, 1 mM EDTA [pH 8.0]) and added to the prehybridized blot. Hybridization was carried out overnight at 65°C. Following hybridization, the blot was washed at 65°C in wash solution (20 mM. Na_2_HPO_4_ [pH 7.2], 1% SDS) and exposed to Kodak X-OMAT film.

LMPCR primer sets for analysis of the upper strand of the *Snrpn* promoter region were:

Primer set 1: TY381 (CACTCCTCAGAACCAAGC) and TY382 (CGGCTCCCTCTCCTCTCTGCGCTA; 64°C); Primer set 2: TY644 (ACTAACACACCCAAGGAGTC) and TY643 (CGGCTCCAAAGGATTGCTCACCAAT; 64.5°C).

These primer sets covered a region on the upper strand from positions −170bp to +125 bp relative to the *Snrpn* transcription initiation site. The LMPCR primer set for analysis of the *Snrpn* promoter region on the lower strand was:

Primer set 3: TY557 (TGATGCTTGCAATCACTTGG) and TY558 (CACGCTCAAATTTCCGTAGTAGGAATGTTC; 61.5°C).

This primer set covered a region on the lower strand from position −125 bp to +100 bp of the *Snurf-Snrpn* promoter region relative to the transcription initiation site. For the control guanine-specific *Snrpn* sequencing ladder, genomic DNA purified from Tg^PWS(del)^ and Tg^AS(del)^ mouse brain tissue was treated with 0.5% DMS at room temperature for 45 seconds, then cleaved with piperidine and subjected to ligation-mediated PCR as described for the experimental samples.

For analysis of *Mkrn3*, DMS treatment was performed using approximately ∼2–3×10^7^ Tg^PWSdel^ or Tg^ASdel^ cells for each DMS treatment. LMPCR was performed essentially as previously described by Chen et. al. [28] with the following modifications. To ensure all amplified fragments had identical 3′ ends, ∼1 unit per reaction of Taq DNA polymerase was added to the final extension step and incubated for 30 min. at 76°C. To cover the immediate *Mkrn3* 5′ flanking region, the following four pairs of gene-specific primer sets were used:

1Ua: CTTCAGCACCTGCCTCC for primer extension, and GTGGGCCTCAATGGGAGCTGTAGACT for LMPCR; 

1Ub: CTCTTCCATCTTACAGCGTG for primer extension, and TGCCTCTAACACTGTCCAAAAACCCGAATA for LMPCR;

1La: ATCCCAGTGTCTCAAGCAG for primer extension, and GAAACAGGCACGCGAAAAACATGGC for LMPCR;

1Lb: ACCTGGAGAGTTTAAAACATCA for primer extension, and TGAGGGGAAACACTGTGGAAACGGG for LMPCR.

PCR products from LMPCR were size-fractionated on DNA sequencing gels, and electrotransferred onto a nylon membrane as described previously [27], [30]. *In vivo* footprints were visualized with ^32^P-labelled strand-specific probes. Hybridization probes were prepared from a double-stranded plasmid template (plasmid EN40 generously provided by Robert Nicholls) containing a 3.9 kb EcoRI-NotI fragment (encompassing the *Mkrn3* promoter region) cloned into a pZErOTM-2 vector. Plasmid DNA was first purified and linearized by double digestion with NotI and EcoRI, then strand-specific radiolabeled single-stranded hybridization probes were synthesized from the linearized plasmid template with the same gene-specific primer used for each LMPCR reaction. For each probe synthesis reaction, 4 µl H_2_O, 3 µl template (∼0.5 ug/ul), and 1 µl of the gene-specific primer (0.3 µg/µl) were first incubated at 95°C for 10 min. and snap frozen on dry ice. Then, 5 µl of 5X labeling buffer from the DECAprimer^TM^II (Ambion, Cat#1455) kit (without dCTP), 10 µl α-^32^P-dCTP (10 µCi/µl, 3000Ci/mmol), and 1 µl DNA pol I Klenow fragment (5 units total; NEB, Cat# M0210S) were added to the denatured template/primer solution and incubated at 37°C for 30 min. The reaction was stopped by adding 35µl formamide-dye solution (1 mg/ml xylene cyanol FF, 1 mg/ml bromophenol blue, 10 mM EDTA, pH 8.0 in formamide) and denatured at 95°C for 10 minutes. The single-stranded radiolabeled probes then were gel-purified by electrophoresis in a 6% acrylamide gel and excised from the gel. Radiolabeled probes were hybridized to electrotransferred membranes to visualize *in vivo* footprints in autoradiograms as described previously [27], [30]. For the control guanine-specific *Mkrn3* sequencing ladder, purified genomic DNA from each of the fibroblast cell lines derived from Tg^PWS(del)^ and Tg^AS(del)^ embryos was DMS and piperidine treated, then subjected to ligation-mediated PCR, as described above.

### Chromatin immunoprecipitation (ChIP) assays

ChIP assays for the *Snrpn* and *Mkrn3* loci were performed as previously described [17]. PCR primers for real-time PCR ChIP analysis of *Snrpn* were as follows:

Primer set 1 (TY1168, 1169): TTGCAAAGTGCCCTCTCTGTCTG, GATGGGGAGATTTTGAAACAGGATG;

Primer set 2 (TY1170, 1171): AGACCCCTGCATTGCGGC, GCATCTCCGGCTCCCTCTCC;

Primer set 3 (TY1172, 1173): TGGTCTGGGGGATGGGAGC, CTGAACACACAAGCCATGGCAAC;

Primer set 4 (TY1174, 1175): AGGGGTCGTGTCGCATGTCA, AATGGGGAGGGGTCTACTGCC;

Primer set 5 (TY1176, 1177): CATTTGGATTCTGCTTTCGACATTC, CAGGGTGGCCATGTTAGGAACTG.

PCR primers used for ChIP assays of *Mkrn3* were as follows:

Region 1 (−1091 to −971): CCTTAGGTTGTGAGACTCCTTCCAATA, TCTTCCTTTCCTCACCCACTAACTTGAA;

Region 2 (−104 to +18): CGGTGAAGCCCTAGGAATGGTGT, GGAGCGAAGTGCATCGATTTTTGT;

Region 3 (+1526 to +1639): GGTGGTGGATCATCAAGCGCA, GGCCAGGCGAAGCACAGAATG;

Region 4 (+4635 to +4743): GAACAGAGGATACTCCCACTAAAACCAA, TGTGCCAAGGGGTTCTTTGTTCA.

PCR primers used for ChIP assays of *Ndn* were as follows:

TY1210: CCTACCACCCTTCTGGCTTCCCA

TY1211: CTCGGTGGAGACCAGCAGAGGAA.

PCR primers used for ChIP assays of *Magel2* were as follows:

TY1212: CGCCCCTCTGAACAATCCACTTG

TY1213: TCACACAGAGTGTCGGCTGGCTC

For ChIP analysis of YY1 and NRF-1 binding to the *Snrpn* locus in cultured cells, a total of at least three independent immunoprecipitations were performed for each antibody, and quantitative real-time PCR with each primer set for each immunoprecipitation was performed in duplicate. ChIP analysis of YY1 and NRF-1 in mouse tissues consisted of two independent immunoprecipitations and duplicate quantitative PCR reactions for each primer set and immunoprecipitated sample. For ChIP analysis of *Mkrn3* for Sp1 and NRF-2 binding in cultured cells, a total of four immunoprecipitations were performed for each antibody, and quantitative real-time PCR with each primer set for each immunoprecipitation was performed in duplicate. *Mkrn3* ChIP analysis of YY1 in cultured cells, and YY1 and NRF-2 analysis in mouse tissues, consisted of two independent immunoprecipitations and duplicate quantitative PCR reactions for each primer set and immunoprecipitated sample. Antibodies for ChIP analyses were obtained from Santa Cruz Biotechnology.

### Chromosome Conformation Capture (3C)

3C assays were performed as described previously [31], [32] with modifications. To prepare each 3C template, 5–6×10^7^ cells, or primary brain cells from 4–6 mouse brains prepared as described above, were resuspended in 45 ml of fresh RPMI-1640 and cross-linked with 1% formaldehyde at room temperature for 10 minutes. Cells were lysed with 20 strokes of a Dounce homogenizer on ice (pestle B; 10 strokes, one-minute pause, 10 strokes). Lysed nuclei were washed as described then resuspended in 7.24 ml of 1X NEBuffer #2 (NEB) and digested with 8,000 U of EcoRI (NEB) at 37°C overnight. Ligation was performed in 160 ml of ligation reaction buffer; for the ligation reaction, the DNA was divided in half, with one half receiving T4 ligase and the other half serving as a non-ligated control which received the same volume of buffer rather than ligase. Then samples were heated to 65°C for 30′, treated with 10 mg Proteinase K (Invitrogen) overnight at 65°C, then an additional 10 mg Proteinase K was added and the samples were incubated for an additional 2 hours at 42°C. Samples were phenol extracted, ethanol precipitated, desalted, redissolved in 1 ml 1X TE buffer, and treated with 10 µg RNase A to yield final 3C templates. Novel ligation products formed via long-rang interactions in vivo were detected by PCR using the following 3C PCR primers: *anchor*, CATTTCCTTAACCTGGTGCTACGATAG; *a*, GTGCCACACAGTTTGAGAACAGGAT; *d*, ATATGTGCATTTGTCGTTTGTGTATGTATC; *e*, CACAGTTAAAACGAACACAATAGTCCCAA. For a 25 ul PCR reaction using 3C primers, 1–2 µl template DNA was mixed with 10 pmol of each primer, 5 nmol of each dATP, dTTP, dGTP, dCTP, 0.625 units of Hotstar Taq DNA polymerase (Qiagen). All PCR reactions were performed with 45 cycles using the following program: 95°C for 15 minutes, followed by 44 cycles of 95°C for 45 seconds, 57°C for 60 seconds, 72°C for 45 seconds, then an elongation of 10 minutes at 72°C. PCR products were run on 1.5% agarose gels, stained with ethidium bromide and photographed. The DNA sequences of the novel ligated fragments were confirmed by sequencing; PCR-amplified DNA fragments were isolated directly from the gel with the Qiagen Gel Purification Kit and ligated into a Topo TA vector (Topo-TA Cloning Kit, Invitrogen). For each PCR product of the 3C assays, at least 6 clones were sequenced to verify that the expected 3C PCR product was amplified.

## Results

We have postulated previously that regulatory factors associated with the PWS-IC on the paternally-inherited chromosome may mediate PWS-IC function [17]. Therefore, we analyzed the 5′ region of the mouse *Snrpn* gene to identify potential regulatory regions and their associated cis- and trans-acting regulatory elements. We first mapped DNase I hypersensitive (DH) sites in the 5′ region of the *Snrpn* gene in primary brain cells. Then selected DH sites were further characterized to identify binding of specific transcription factors to sites within these DH regions. Distal paternally-expressed genes in the AS/PWS domain regulated by the PWS-IC (e.g., *Mkrn3*, *Ndn*) were also examined for association with specific transcription factors and physical proximity to the PWS-IC to provide insight into potential mechanisms of PWS-IC function.

### DNase I hypersensitivity analysis of the PWS-IC

**Figure 1:**
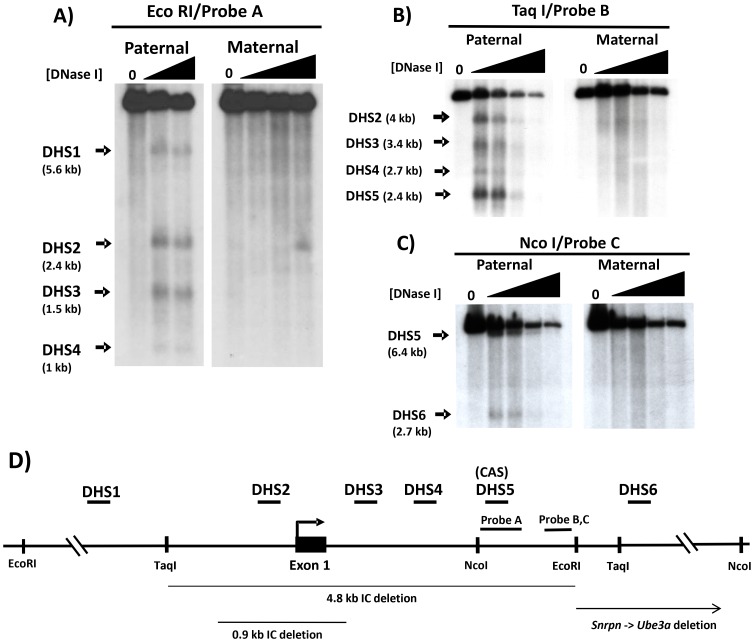
DNase I hypersensitivity of the murine PWS-IC. DNase hypersensitive sites (DHS) were mapped by Southern blotting and indirect end-labeling after DNase I treatment of primary brain cells. The maternal and paternal alleles were analyzed separately using brain cells prepared from mice carrying a 35 kb PWS-IC deletion on either the paternal or maternal chromosome, respectively [8], [18]. Cells were treated with increasing concentrations of DNase I; *0 DNase* samples were purified genomic DNA from untreated brain cells. (A) Analysis by EcoRI digestion and hybridization with probe A. The thick arrows indicate the positions and sizes of prominent reproducible DNase I hypersensitive bands. *Paternal* indicates samples carrying a maternal PWS-IC deletion; *Maternal* indicates samples carrying a paternal PWS-C deletion. B) Analysis by Taq I digestion and hybridization with probe B. C) Analysis by Nco I digestion and hybridization with probe C. D) Summary of DNase I hypersensitive sites and their locations. The diagram depicts the *Snrpn* 5′ region showing relevant restriction enzyme sites and the positions of hybridization probes A, B and C (probe B is contained within probe C). The relative positions of DHS1-6 are indicated by short horizontal bars above the gene; all prominent DH sites (DHS1-DS6) are specific to the paternal chromosome. *CAS* denotes the conserved activator sequence [17] that co-localizes with DHS5. Thin horizontal lines below the gene depict regions deleted by targeted knockouts of the *Snrpn* locus [38], [39]. The bent arrow depicts the location of the transcription initiation site. The position of each DH site relative to the transcription initiation site is estimated to be: DHS1, −3.2 kb; DHS2 includes the transcription initiation site; DHS3, +0.8 kb; DHS4, +1.4 kb; DHS5, +1.7 kb; DHS6, +4.1 kb; the positions of DHS1-DHS6 in the *Snrpn* gene were calculated as an average of at least two independent experiments and/or different restriction enzymes and probes. The same DH sites mapped by different experiments, restriction enzymes, and/or blots generally localized within 200–300 bp of each other, a variation within the range expected by estimating positions derived from band sizes in Southern blots.

Because *Snrpn* and other genes in the AS/PWS region expressed exclusively from the paternally-inherited chromosome are generally expressed at elevated levels in the brain [22], [33], [34], [35], [36], [37], we examined primary brain cells from one day old newborn pups for DH sites. To perform parent-of-origin-specific mapping of DH sites, mice carrying the 35 kb PWS-IC deletion on either the maternally- or paternally-inherited chromosome were used as sources of brain tissue [8], [18]. To identify DH sites across the 5′ region of the *Snrpn* locus, from position −6346 to +2373 (relative to the transcription initiation site), primary brain cells were subjected to DNase I treatment and Southern blotting using EcoRI and hybridization probe A. Results of the EcoRI Southern blot analysis are shown in Fig. 1A; for cells carrying a maternal PWS-IC deletion, in addition to the expected genomic EcoRI fragment, multiple prominent DNase hypersensitive sub-bands were detected at 5.6 kb (DHS1), 2.4 kb (DHS2), 1.5 kb (DHS3), and 1 kb (DHS4) specifically on the paternal chromosome. DHS2 corresponds to the *Snrpn* promoter region, and DHS3 marks the approximate 3′ edge of the differentially methylated 5′ CpG island that starts at the *Snrpn* promoter and spans exon 1 and part of intron 1. In contrast, no prominent hypersensitive bands were reproducibly detected in brain cells carrying a paternal PWS-IC deletion, though numerous faint bands could be detected in some EcoRI blots as shown in Fig. 1A. Thus, the major hypersensitive bands detected by EcoRI and probe A were all specific to the paternally-inherited allele.

To confirm DH sites detected by EcoRI digestion, DNase-treated samples were digested with TaqI and the Southern blot was hybridized with probe B (Figure 1B). In addition to the normal genomic TaqI band, Southern blot analysis revealed four hypersensitive sub-bands 4.0 kb, 3.4 kb, 2.7 kb, and 2.4 kb in size exclusively on the paternal allele, corresponding to hypersensitive sites DHS2, DHS3, DHS4, and DHS5, respectively. DHS2-4 mapped by TaqI/probe B within 200–300 bp of their positions as determined by EcoRI/probeA, within the range expected for mapping of DH sites by Southern blot analysis. Both DHS2 and DHS5 correspond to conserved DH sites that were also detected in the *SNPRN* gene in human lymphoblasts [12], [17]. DHS5 corresponds to the position of the murine conserved activator sequence (CAS), a conserved sequence located ∼1.7 kb downstream of the transcription initiation site that was identified as an enhancer in the human *SNRPN* gene [17]. To detect DH sites downstream of the CAS in the first intron of *Snrpn*, further analysis was performed using NcoI and hybridization probe C. As shown in Fig. 1C, DNase hypersensitive bands in NcoI digests were detected at 6.4 kb and 2.7 kb only on the paternal chromosome, corresponding to DHS5 and DHS6, respectively.

We now have identified and determined the allele-specificity of six major DH sites in the 5′ region of the murine *Snrpn* gene in primary brain cells, all of which are specific to the paternal chromosome. Based on the sizes of the hypersensitive bands and the positions of the restriction sites used to detect them, the positions of these DH sites relative to the *Snrpn* gene are summarized in Fig. 1D. DHS1-DHS5 are all located within the 6 kb interval that defines the minimal murine PWS-IC [19]; DHS6 is located ∼1.8 kb downstream of the minimal PWS-IC. We propose that these DH sites, and the cis- and trans-acting regulatory elements contained therein, are major functional components of the murine PWS-IC. And as detailed in the Discussion section, the position of these DH sites in the PWS-IC provides potential explanations for the phenotypes reported for various targeted deletions of the murine *Snrpn* gene [38], [39].

### *In vivo* footprint analysis of the *Snrpn* promoter region

To identify specific regulatory sites and factors within these major paternal DH sites, we first analyzed the *Snrpn* promoter region (DHS2) by performing ligation-mediated PCR (LMPCR) *in vivo* footprint analysis on primary mouse brain cells. To examine the maternal and paternal alleles independently, *in vivo* footprinting analysis was performed on brain cells isolated from Tg^PWS(del)^ and Tg^AS(del)^ mice that carry a deletion of the entire AS/PWS domain on either the paternally-inherited or maternally-inherited chromosome, respectively [20]. Sequence-specific DNA-protein interactions at individual guanine residues (DMS specifically modifies guanines) are detected as gel bands of increased or decreased intensity (compared to a control sample of purified genomic DNA treated with DMS and subjected to LMPCR) due to increased or decreased reactivity to DMS.

**Figure 2:**
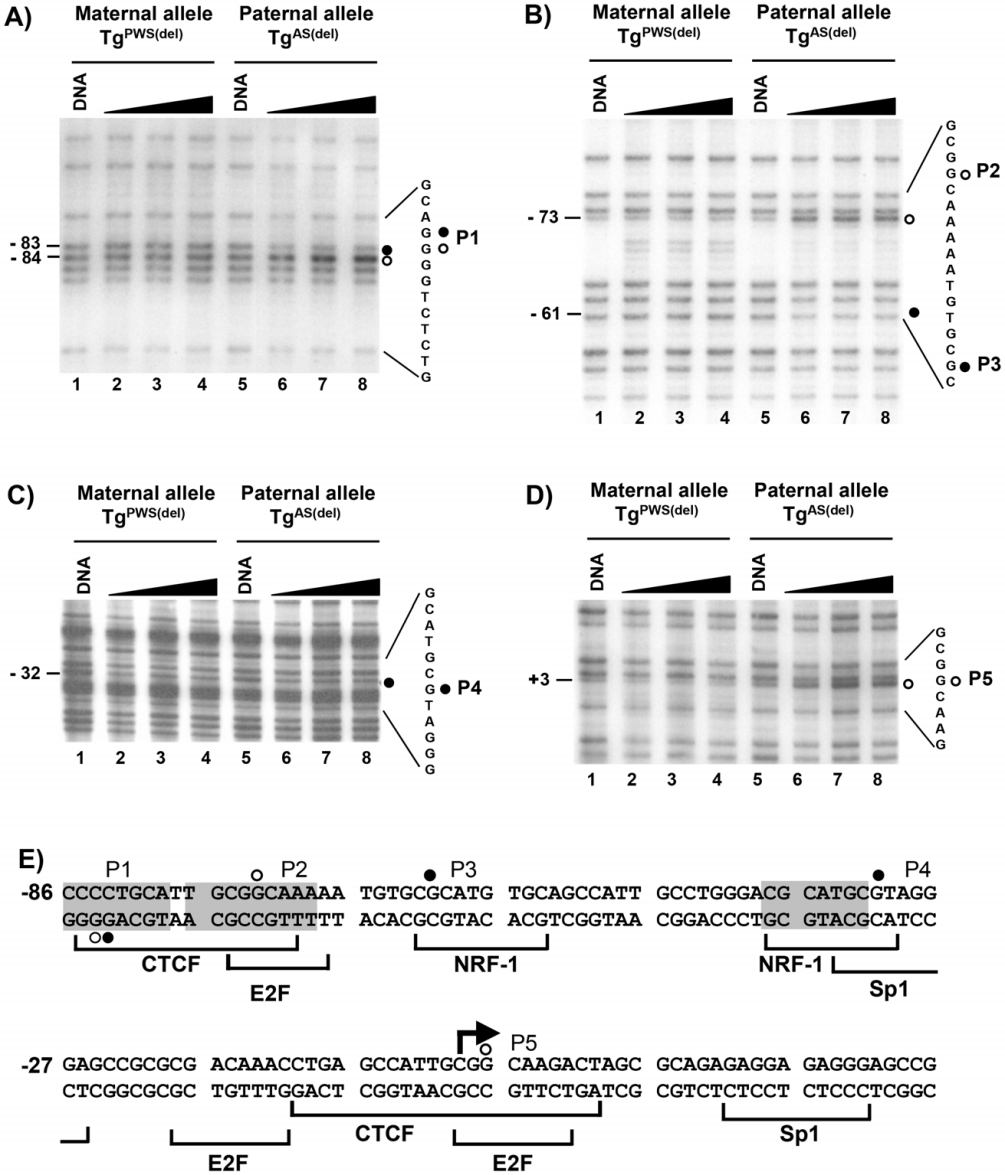
*In vivo* footprint analysis of the *Snrpn* promoter region. LMPCR *in vivo* footprinting with dimethyl sulfate (DMS) was performed on primary brain cells isolated from Tg^PWS(del)^ and Tg^AS(del)^ mice carrying a deletion of the entire murine AS/PWS region on either the paternal or maternal chromosomes, respectively [20]. Cells were treated with increasing time of exposure to DMS. “DNA” represents control samples of purified genomic DNA subjected to DMS treatment and LMPCR in parallel with samples treated *in vivo* with increasing time of exposure to DMS (60, 90, 120 seconds). The LMPCR primer sets detecting each footprint are described in the text. A) – D) Autoradiograms showing *in vivo* footprints P1 – P5 in the *Snrpn* promoter region. Filled circles represent footprints detected as guanines protected from DMS reactivity, open circles represent footprints at guanine sites showing enhanced DMS reactivity. Numbers on the left of each sequencing gel denote the location of each footprint relative to the transcription initiation site. The nucleotide sequences to the right of each autoradiogram show the DNA sequence containing and flanking each footprint; the filled/open circles indicate the location of each *in vivo* footprint within the DNA sequence and correspond to the footprinted sites shown on the left of each autoradiogram. E) Summary of *in vivo* footprints in the *Snprn* promoter region of the paternal allele. The footprinted sites detected by LMPCR *in vivo* footprinting are shown within the nucleotide sequence of the promoter region of the mouse *Snprn* gene. Open and filled circles denote footprinted sites as described above. At each footprinted position, the footprinted site corresponds to the strand containing the guanine nucleotide. Shaded nucleotide sequences indicate sequences conserved between the human and mouse *Snrpn* promoters. Brackets below the nucleotide sequence indicate the location and identity of potential transcription factor binding sites. The bent arrow depicts the transcription initiation site; numbering of nucleotides is relative to the transcription initiation site.

Figures 2A, B, C, D show representative sequencing gels for the *in vivo* footprint analysis in mouse brain cells. All of the footprints identified lie within a 100 bp region which overlaps the *Snrpn* minimal promoter defined by Hershko et al. in transient expression assays [40]. As shown in Figure 2A, analysis of the lower strand with primer set 3 identified footprint P1 on the paternal allele as a band of decreased intensity at position −83 and a band of increased intensity at position −84 on the *in vivo* treated samples of intact Tg^AS(del)^ cells (lanes 6–8) relative to naked DNA from Tg^AS(del)^ cells (lane 5). This altered pattern of band intensity was not observed on the maternal allele (lanes 1–4) in Tg^PWS(del)^ cells, which indicates that footprint P1 is specific to the paternal allele. Analysis of the upper strand with primer set 1 (Figure 2B) identified footprint P2 at position −73 as a gel band of increased intensity (lanes 6–8) compared to naked DNA (lane 5) only on the paternal allele (i.e., in Tg^AS(del)^ cells). A second footprint specific to the paternal allele, P3, was detected with this primer set at position −61 as a band of decreased intensity (lanes 6–8) relative to naked DNA (lane 5). Neither footprint was detected in Tg^PWS(del)^ cells carrying the maternal allele (lanes 1–4). Figures 2C and 2D show footprints P4 at position −32 and P5 at position +3 on the upper strand of the paternal allele using primer set 2. For footprint P4 (Fig. 2C), the band at position −32 was slightly less intense than the band above (at position −34) in samples from cells treated I vivo with DMS (lanes 6–8); this is in contrast to the bands at positions −32 and −34 in samples of naked DNA from Tg^AS(del)^ cells (lane 5) and in samples containing the maternal allele in Tg^PWS(del)^ cells which were of the same intensity. This footprint (P4) was subtle in comparison to other footprinted sites, but was highly reproducible in multiple independent sequencing gels, LMPCR reactions, and preparations of DMS-treated samples. Footprint P5 (Fig. 2D) was detected specifically on the paternal allele as a band of increased relative intensity at position +3 in the *in vivo* treated samples (lanes 6–8) of Tg^AS(del)^ cells relative to naked DNA from the same cells (lane 5).

Figure 2E summarizes the *in vivo* DMS footprint pattern for the *Snrpn* promoter on the paternal allele and shows potential transcription factor binding sites that were identified by analysis with the TRANSFAC transcription factor database. The sequence associated with P1 is a potential binding site for the transcription factor CTCF which is a ubiquitous transcription factor involved in numerous mechanisms of gene regulation, including the regulation of other imprinted gene clusters such as the *H19* and *Igf2* cluster and long-range organization of the genome [41]. Furthermore, the potential CTCF binding site associated with footprint P1 is conserved between the human and mouse promoters. Previous analysis of the human *SNRPN* promoter showed several potential CTCF binding sites, two of which were also associated with DMS *in vivo* footprints; however, ChIP assays were unable to confirm binding of CTCF to the human promoter [17]. Footprints P2 and P5 both lie within a repeated sequence of 11 nucleotides (cattgcggcaa) that coincides with potential E2F binding sites and partially overlap with potential CTCF binding sites; P2 is also contained within a sequence that is conserved between the human and mouse *SNRPN* promoters. Footprints P3 and P4 lie within potential binding sites for nuclear respiratory factor-1 (NRF-1), which also has been shown to be associated with the human *SNRPN* locus [17]. NRF-1 is a transcription factor commonly associated with the activation of nuclear-encoded genes involved in mitochondrial biosynthesis and respiration [42]. As with the human *SNRPN* promoter region, no evidence for binding of YY1 was detected by *in vivo* footprinting in the minimal mouse *Snrpn* promoter.

A similar DMS *in vivo* footprinting analysis was performed on spleen cells isolated from Tg^PWS(del)^ and Tg^AS(del)^ mice. However, in contrast to our results in brain cells, no *in vivo* footprints were detected in the promoter region in spleen cells (data not shown).

### Analysis of *Snrpn* transcription factor binding by ChIP

To confirm *in vivo* footprint analysis for binding of NRF-1 and YY1 to the *Snrpn* promoter region (DHS2), we performed ChIP analysis using antibodies against NRF-1 and YY1. ChIP assays for NRF-1 and YY1 were also performed on the murine CAS (i.e., DHS5) because the human CAS was shown to be bound by NRF-1 and YY1 on the paternal allele [17]. In addition, ChIP assays were directed at a potential YY1 binding site associated with DHS6 located ∼4.2 kb downstream of the *Snrpn* transcription initiation site. ChIP analyses focused on NRF-1 and YY1 because: 1) NRF's and YY1 bind to other paternally expressed genes in the AS/PWS domain (see below), 2) binding of nuclear respiratory factors suggest a potential relationship with regulation of respiration, and 3) YY1 binding sites have been reported to be associated with regulatory regions of other imprinted loci [43], [44].

**Figure 3:**
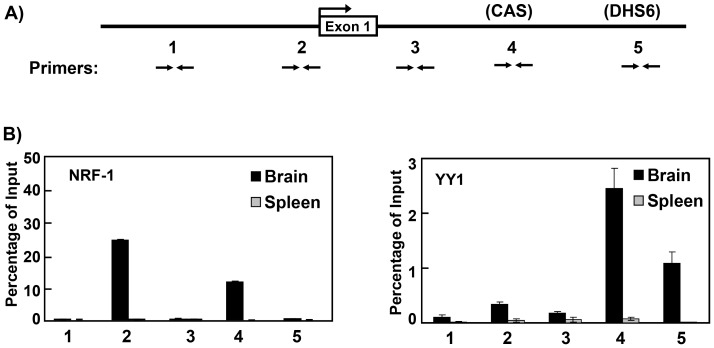
ChIP analysis of transcription factor binding to the *Snrpn* locus. Chromatin immunoprecipitation analysis was performed to assay for binding of NRF-1 and YY1 to DH sites in the *Snrpn* 5′ region in primary brain and spleen cells of C57Bl/6 mice. A) Diagram of the mouse *Snrpn* 5′ region showing the regions assayed by ChIP. Pairs of opposing horizontal arrows depict the location of PCR primer sets used for real-time PCR in ChIP assays. Primer sets 1 and 3 are negative controls and amplify regions where no known factors are thought to be bound. B) Results of ChIP assays performed with antibodies against the transcription factors NRF-1 and YY1. Black and gray vertical bars denote the results for the ChIP experiments on brain cells and spleen cells of C57B/6 mice, respectively. Numbers under the graphs indicate the results obtained for the corresponding PCR primer sets shown in panel A.

As indicated in Figure 3A, ChIP assays were performed using PCR primers specific to a site ∼1 kb upstream of the transcription initiation site (primer set 1), within the *Snrpn* promoter region (primer set 2), a region ∼900 bp downstream of the transcription initiation site (primer set 3), within the murine CAS sequence (primer set 4), and the potential YY1 binding site at DHS6 (primer set 5). ChIP assays were performed on primary newborn brain and adult spleen cells prepared from C57Bl/6 mice. As shown in Figure 3B, ChIP analysis revealed NRF-1 binding in the *Snrpn* promoter region as well as in the mouse CAS in brain cells. No evidence for binding of NRF-1 was detected using primer sets 1, 3, or 5 in brain cells. ChIP analysis detected binding of YY1 in the region the CAS as well as the potential YY1 binding site at DHS6 in brain cells, but not in the *Snrpn* promoter or regions associated with primers 1 or 3. Because these ChIP assays were performed on brain cells from C57Bl/6 mice, it was not possible to determine which allele(s) was bound by NRF-1 and YY1. Nonetheless, because *Snrpn* is expressed only from the paternally-inherited allele, all *in vivo* footprints in the promoter region were detected only on the paternal allele, and the binding of NRF-1 and YY1 in the human *SNRPN* CAS occurred only on the paternal allele, it is very likely that the results from these ChIP assays reflect binding of NRF-1 and YY1 specifically on the paternal allele in the *Snprn* locus in brain cells. ChIP assays using antibodies against CTCF detected little or no binding of CTCF within the promoter region in brain cells (data not shown), consistent with previous ChIP analysis of the human *SNRPN* promoter region in lymphoblasts which also showed no detectable binding of CTCF though the human promoter also exhibited *in vivo* footprints at or adjacent to potential CTCF sites [17]. ChIP analysis of spleen cells from C57Bl/6 mice (Fig 3B) did not detect association of either NRF-1 or YY1 with any of the primer sets used, consistent with our *in vivo* footprint analysis of the minimal *Snrpn* promoter region in spleen cells which detected no *in vivo* footprints on either the paternal or maternal alleles.

### DNase I hypersensitivity analysis of the *Mkrn3* locus

To identify cis- and trans-acting regulatory elements in a distal gene expressed specifically from the paternally-inherited chromosome and regulated by the PWS-IC, we analyzed transcription factor binding to the murine *Mkrn3* locus. This allowed us to compare regulatory elements between the murine PWS-IC region (and *Snrpn* gene) and one of its target genes. We first determined the location of DH sites in the *Mkrn3* locus in Tg^PWSdel^ and Tg^ASdel^ cells, immortalized cultured fibroblast cell lines derived from the mouse model of PWS in which a transgene insertion led to deletion of the AS/PWS region (the mouse line termed Tg^PWS/AS(del)^; [20]). Imprinting in the AS/PWS region is maintained in this mouse line, with paternal inheritance of the deletion yielding pups that failed to thrive and died within one week, and maternal inheritance of the deletion showing a mild phenotype that includes late onset obesity [20], [45]. Cultured Tg^PWSdel^ cells derived from these mice in which the deletion is paternally inherited allows for analysis of the maternal allele only, whereas Tg^ASdel^ cells in which the deletion is maternally inherited allows for analysis of the paternal allele only. To verify that DNA methylation imprints in the *Mkrn3* locus are maintained in cultured Tg^ASdel^ and Tg^PWSdel^ fibroblast cells, the *Mkrn3* promoter region in these cells was subjected to high resolution DNA methylation analysis by sodium bisulfite genomic sequencing. Analysis of the 5′ CpG island, including the promoter region, from positions −131 to +162 showed hypermethylation of the maternal allele in Tg^PWSdel^ cells and an essentially unmethylated paternal promoter in Tg^ASdel^ cells (data not shown). Thus, the expected differential DNA methylation imprints are present at the *Mkrn3* locus in the immortalized cultured fibroblasts derived from the Tg^PWSdel^ and Tg^ASdel^ mouse lines. Maintenance of imprinting in the Tg^PWSdel^ and Tg^ASdel^ cell lines also was verified by analysis of allele-specific gene expression within the AS/PWS domain (data not shown). Thus, the *Mkrn3* locus is correctly imprinted in the Tg^PWSdel^ and Tg^ASdel^ cell lines and that these lines can serve as a cell culture system to examine aspects of imprinted regulation of the *Mkrn3* gene.

**Figure 4:**
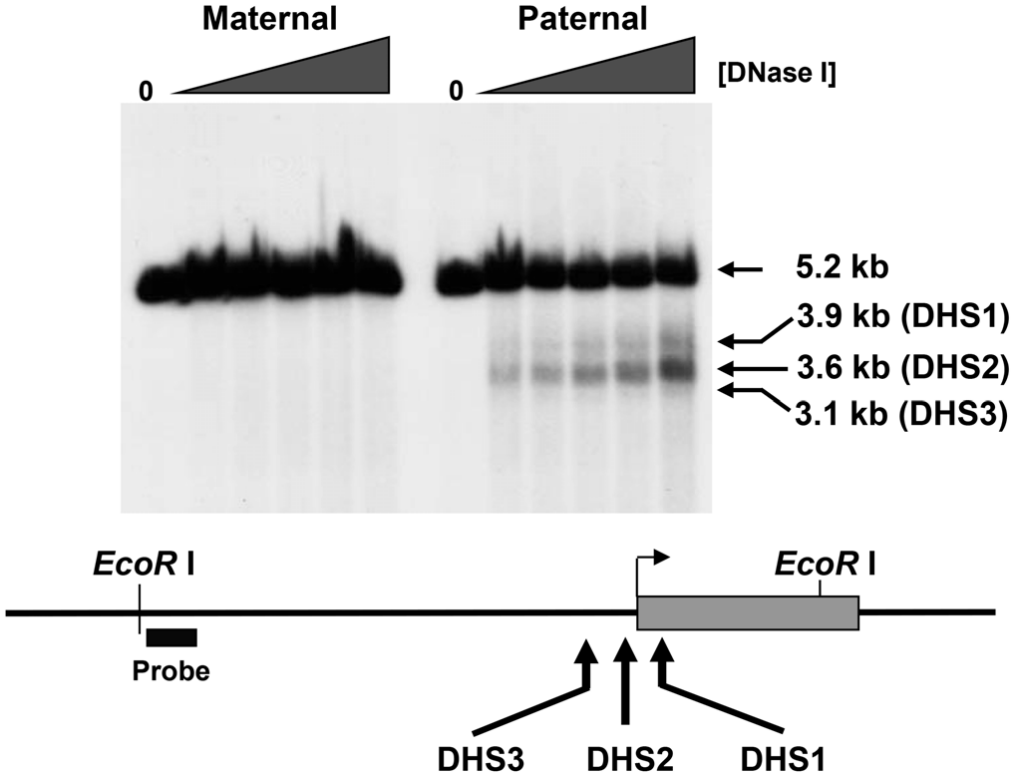
Mapping of DNase I hypersensitive sites in the *Mkrn3* locus. Intact cells were treated with increasing concentrations of DNase I and purified genomic DNA was analyzed by indirect end-labeling by Southern blotting as described in Materials and Methods. Maternal and paternal alleles were analyzed separately using Tg^PWSdel^ (PWS) and Tg^ASdel^ (AS) fibroblasts. Lanes marked “0” represent samples of purified mouse genomic DNA from Tg^PWSdel^ or Tg^ASdel^ cells. “Probe” indicates location of hybridization probe used in Southern blot analysis.

As shown in Figure 4, in Tg^PWSdel^ cells which contain the maternally-inherited allele, only the expected 5.2 kb genomic EcoRI DNA fragment was detected. In contrast, three subfragments generated by DNase hypersensitivity (at 3.1 kb, 3.6 kb and 3.9 kb) specific to the paternally-inherited allele were detected in Tg^ASdel^ cells. The strongest DNase-dependent subfragment, representing DH site 2 (DHS2), was centered ∼100 bp upstream of the transcription initiation site of *Mkrn3*, whereas DHS1 mapped ∼190 bp downstream of the transcription initiation site. The weakest hypersensitive site, DHS3, was located ∼600 bp upstream of the transcription initiation site. Because DHS2 was the strongest DH site in our analysis and localized to the *Mkrn3* promoter, all subsequent studies were focused on this region.

### *In vivo* footprint analysis of the *Mkrn3* promoter region

DNA sequence analysis using TRANSFAC for the presence of potential transcription factor binding sites within DHS2 revealed binding sites for various factors including multiple nuclear respiratory factor-2 (NRF-2), YY1, and Sp1 sites, as well as a binding site for STAT1. Like NRF-1, NRF-2 is a transcription factor commonly found as an activator of nuclear genes associated with mitochondrial biosynthesis and oxidative phosphorylation [42]. To determine whether or not these sites were occupied *in vivo*, LMPCR *in vivo* footprinting was performed across DHS2 (i.e., the promoter region) from positions −200 to +150 on both the upper and lower strands using DMS. Four LMPCR primer sets were used, 1Ua and 1Ub to footprint the upper strand, and 1La and 1Lb to footprint the lower strand. Cell lines Tg^PWSdel^ and Tg^ASdel^ were used to footprint the maternal and paternal alleles, respectively, as described above.

**Figure 5:**
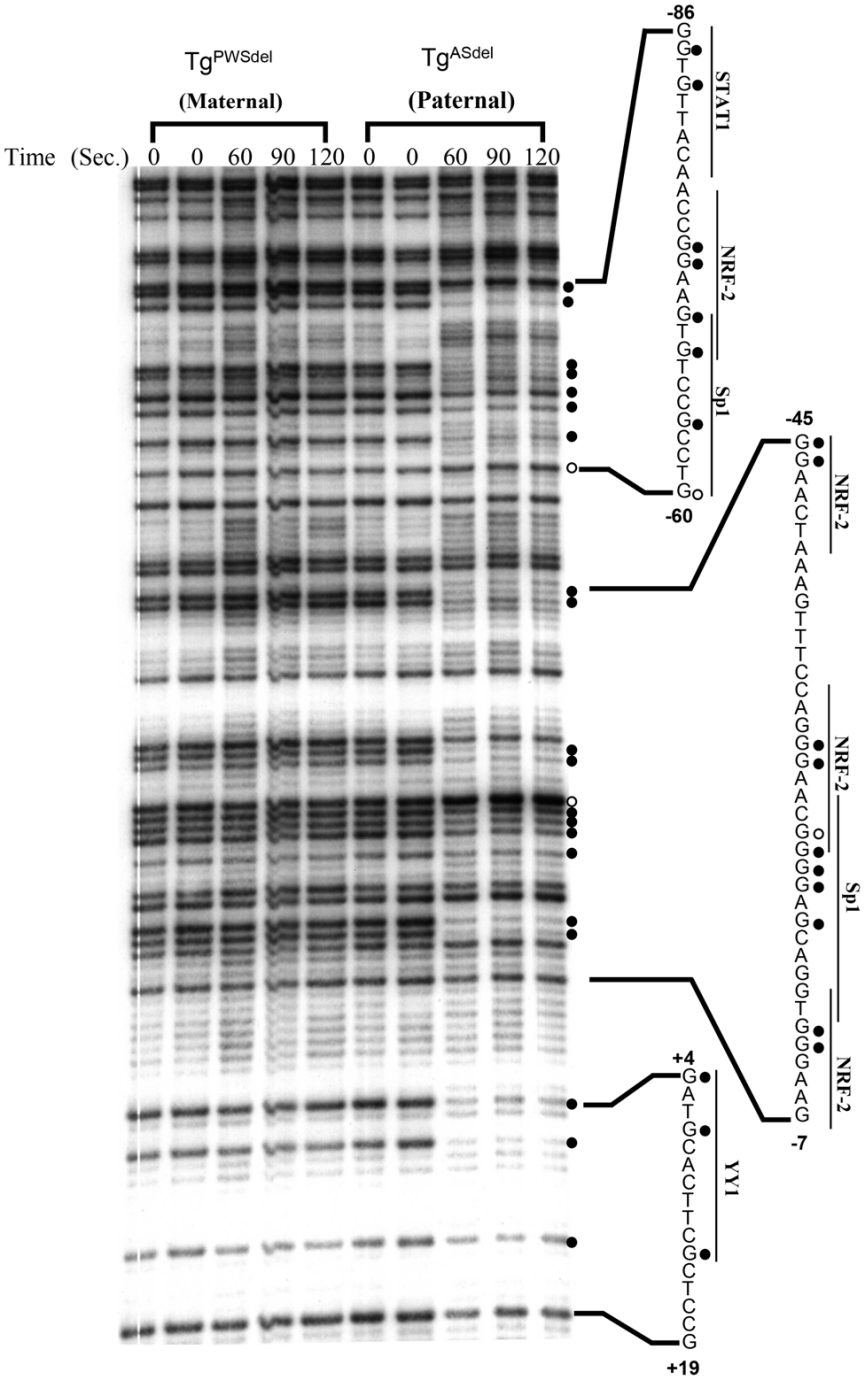
*In vivo* footprint analysis of the 5′ region using primer set 1Ua. A representative autoradiogram is shown for analysis of the upper strand spanning positions −86 to +19. Maternal and paternal alleles were analyzed separately using cultured Tg^PWSdel^ and Tg^ASdel^ cells, respectively. Intact cells were treated with dimethyl sulfate (DMS) for 60, 90, and 120 seconds. Time 0 represents control purified genomic DNA samples from Tg^PWSdel^ and Tg^ASdel^ cells which were treated in vitro with DMS/piperdine, then subjected to LMPCR to generate a guanine-specific sequencing ladder of the region. *In vivo* footprinted sites on the paternal allele are indicated on the right of the sequencing gel with closed circles representing DMS-protected guanine residues and open circles representing sites of enhanced DMS reactivity *in vivo*. Nucleotide sequences containing footprinted sites are shown to the right of the autoradiogram; numbers represent positions relative to the *Mkrn3* transcriptional initiation site. Potential transcriptional factor binding sites as identified in the Transfac database are indicated beside the corresponding nucleotide sequences.

Figure 5 shows a representative autoradiogram of the LMPCR *in vivo* footprinting pattern of the upper strand from positions −86 to +19 using LMPCR primer set 1Ua. Comparison of the DMS modification and cleavage patterns between *in vivo*-treated samples (for 60, 90, and 120 sec.) and naked DNA treated with DMS (lanes 0) revealed footprinted sites only on the paternal allele (i.e., in the Tg^ASdel^ cell line); no consistent footprints were observed in this region on the maternal allele (in Tg^PWSdel^ cells). Sites of DMS protection or enhanced DMS reactivity were clearly detected at guanines associated with potential binding sites for STAT1 (positions −85, −83), overlapping binding sequences for NRF-2 and Sp1 (−74, −73, −70, −68, −64, −60), NRF-2 (−45, −44, −28, −27, −12, −11), overlapping NRF-2 and Sp1 binding sites (−23, −22), and Sp1 (−21, −20, −18). Footprinted guanine nucleotides at positions +4, +7, and +14 on the paternal allele located just downstream of the primary transcription initiation site are associated with a sequence that bears a resemblance to a binding site for YY1 but is not an absolute match for the consensus YY1 binding sequence. No other footprints were detected on the upper strand in the region from −120 to +46 using primer set 1Ua in either the Tg^ASdel^ or Tg^PWSdel^ cells.

**Figure 6:**
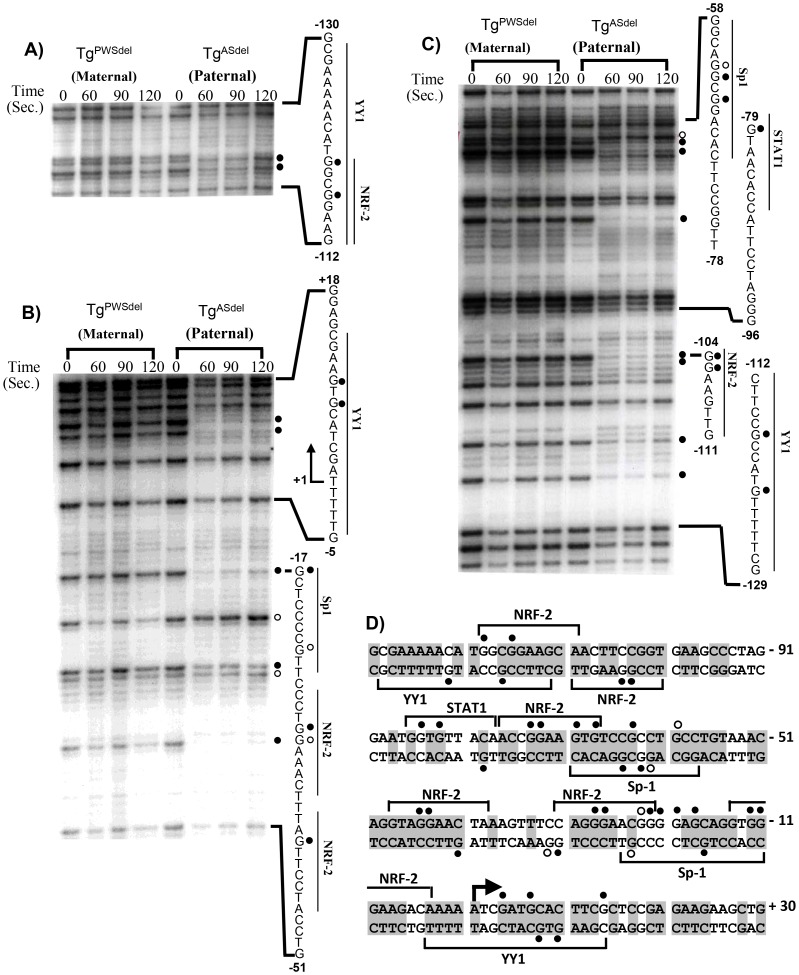
*In vivo* footprint analysis using primers 1Ub, 1La, and 1Lb. A) Primer set 1Ub. A representative autoradiogram is shown depicting analysis of the upper strand from positions −112 to −130. All symbols and designations are identical to those described for Fig. 5. B) Primer set 1La. A representative autoradiogram is shown depicting analysis of the lower strand from positions −51 to +18. All symbols and designations are identical to those described for Fig. 5. The bent arrow represents the transcription initiation site. C) Primer set 1Lb. A representative autoradiogram is shown depicting analysis of the lower strand spanning positions −129 to −58. All symbols and designations are identical to those described for Fig. 5. D) Summary of *in vivo* footprints on the paternal allele in the 5′ region of *Mkrn3*. Solid circles represent DMS-protected guanine residues and open circles represent sites of enhanced DMS reactivity *in vivo*; footprinted sites depicted above the nucleotide sequence were detected on the upper strand, and footprinted sites shown below the nucleotide sequence were detected on the lower strand. No footprints were detected on the maternal allele. Numbers indicate positions relative to the transcription initiation site. Potential transcriptional factor binding sites as identified by the Transfac database are indicated as brackets above or below the corresponding nucleotide sequence. Nucleotides that are conserved between the mouse, rat, and human *Mkrn3* promoter sequences are indicated by shaded boxes.

To examine the *Mkrn3* promoter region further upstream of position −120 on the upper stand, a second primer set, 1Ub, was used to detect footprints in the region from positions −50 to −220 (Fig. 6A). In addition to footprints also detected with primer set 1Ua described above, primer set 1Ub revealed new DMS-protected footprints at positions −116 and −119 on the paternal allele associated with overlapping binding sites for NRF-2 and YY1.

To analyze DNA-protein interactions on the lower strand, primer set 1La assayed for footprints in the approximate region between positions −51 to +109, and primer set 1Lb assayed the approximate region between positions −191 to −24. As with footprints on the upper strand, all footprints detected by these primers on the lower strand were specific to the paternal allele. Paternal-specific *in vivo* footprints detected by primer set 1La are shown in Figure 6B. Two footprinted guanines at positions +10 and +8 were associated with a potential YY1 binding site, and a DMS-protected guanine at position −17 and a guanine with enhanced DMS reactivity at position −24 were associated with a potential Sp1 binding site. Footprinted sites at positions −31 and −32, as well as position −41, were associated with two adjacent NRF-2 binding sites. Analysis using primer 1Lb (Fig. 6C) showed paternal-specific footprints at −62, −63, and −65, all within a sequence corresponding to an Sp1 binding site. In addition, a DMS-protected guanine at position −79 is contained within a potential STAT1 binding site, footprints at positions −104 and −105 correspond to nucleotides within the core binding sequence for NRF-2, and footprints at positions −117, and −122 are located within a YY1 binding site.

Figure 6D shows the nucleotide sequence of the mouse *Mkrn3* promoter region and a summary of the *in vivo* footprint pattern determined by this study. Most of the *in vivo* footprints were robust and readily detectable, and all footprints were specific to the paternally-inherited allele; no evidence for any other footprints in the region spanning positions −200 to +150 was detected on either strand of the paternally- or maternally-inherited alleles. The majority of the footprinted sites on one strand showed a corresponding footprinted site(s) across or nearby on the opposite strand, thereby confirming the presence of most footprints in the region. All of the footprinted sites in this 350 bp region are located within or adjacent to binding sites for the transcription factors NRF-2, Sp1, YY1, or STAT1 and generally in regions of the promoter that are strongly conserved in the mouse, rat, and human *Mrkn3* promoter sequences. Our finding of *in vivo* footprints at potential YY1 and NRF-2 binding sites in the *Mkrn3* locus indicate a notable similarity of transcription factors interacting with both *Mkrn3* and the PWS-IC/*Snprn* gene.

### Analysis of *Mkrn3* by ChIP

**Figure 7:**
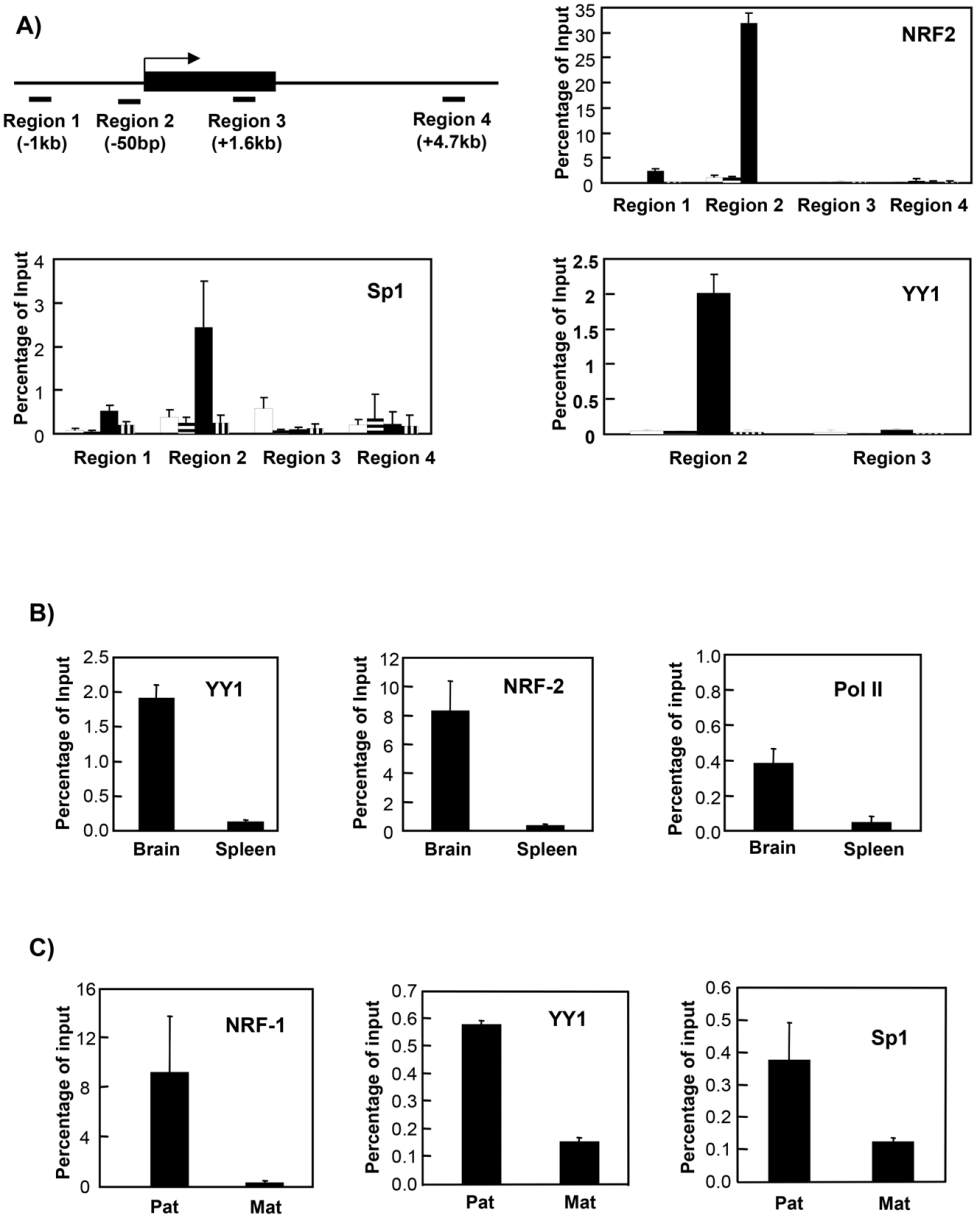
ChIP analysis of the *Mkrn3* and *Ndn* promoters. A) ChIP analysis of the *Mkrn3* locus. Antibodies against NRF-2, Sp1, and YY1 were used to immunoprecipitate chromatin from the maternal and paternal alleles separately in Tg^PWSdel^ and Tg^ASdel^ mouse fibroblasts, respectively. The location of primers used to examine transcription factor binding within regions 1–4 across the *Mkrn3* locus are described further in the main text. The solid rectangle depicts the intronless *Mkrn3* gene; the bent arrow represents the transcription initiation site. Open bars represent analysis of the maternal allele, bars with horizontal stripes represent control samples from Tg^PWSdel^ cells (maternal allele) treated with no antibody, solid bars represent analysis of the paternal allele, and bars with vertical stripes represent control samples from Tg^ASdel^ cells (paternal allele) treated with no antibody. B) ChIP analysis of the *Mkrn3* promoter region (region 2 in panel A) in primary mouse brain and spleen cells. Brain and spleen cell preparations from C57BL/6 mice were subjected to ChIP analysis with antibodies against YY1, NRF-2, or RNA polymerase II. C) ChIP analysis of the *Ndn* promoter region in Tg^PWSdel^ and Tg^ASdel^ cells using antibodies against NRF-1, YY1, and Sp1.

To verify results of the *in vivo* footprinting studies that suggested binding of YY1, Sp1, and NRF-2 in the *Mkrn3* promoter, ChIP analysis was performed at four sites across the *Mkrn3* locus (Fig. 7A) in Tg^ASdel^ or Tg^PWSdel^ cells: ∼1 kb upstream of the transcription initiation site (region 1), in the immediate 5′ flanking region from positions −105 to −5 (region 2), within the body of the gene from positions +1639 to +1526 (region 3), and ∼4.7 kb downstream of the transcription initiation site (region 4). Figure 7A shows results of ChIP analysis across the *Mkrn3* locus using antibodies against NRF-2, Sp1, and YY1. NRF-2 is highly enriched in the promoter region on the paternal allele (i.e., only in the Tg^ASdel^ cells), with no other regions assayed within or flanking *Mkrn3* showing significant levels of NRF-2 binding. This result is consistent with *in vivo* footprinting data that indicate transcription factor occupancy at multiple potential NRF-2 binding sites (Fig. 6D). Similar ChIP results (Fig. 7A) are demonstrated for Sp1 and YY1; interaction of both Sp1 and YY1 occurs in the promoter region of *Mkrn3* exclusively on the paternal allele, again consistent with the *in vivo* footprint patterns shown above (Fig. 6D).

To verify that transcription factor binding to the *Mkrn3* promoter observed in a cultured cell line (Tg^ASdel^) reflected a similar pattern of factor binding within normal intact mouse tissues, ChIP analysis was performed on primary brain and spleen cell preparations from C57BL/6 mice. As shown in Figure 7B, interaction of both YY1 and NRF-2 with the *Mkrn3* promoter region (i.e., region 2 in Fig. 7A) occurs at significantly higher levels in brain cells as compared to spleen cells. Thus, ChIP analysis of the *Mkrn3* promoter in mouse brain and spleen tissues demonstrated tissue-specific binding of YY1 and NRF-2, and also indicated that interaction of YY1 and NRF-2 with the *Mkrn3* promoter is not unique to the immortalized Tg^PWSdel^ fibroblast cells but also occurs within primary brain tissue. Though ChIP analysis of *Mkrn3* in tissues from C57BL/6 mice did not allow us to determine allele-specific interactions of these transcription factors with the *Mkrn3* promoter, it is highly likely that the interaction of YY1 and NRF-2 in brain cells was primarily (if not entirely) on the paternal allele because the *Mkrn3* gene has been shown to be paternally expressed in mouse brain tissue [22]. The low level of RNA polymerase II association with the *Mkrn3* promoter in spleen cells compared to brain cells (Fig. 7B) reflects the tissue-specific expression pattern of *Mkrn3* and is consistent with the low levels of YY1 and NRF-2 binding in spleen cells.

To examine the possibility that similar transcription factors may also be associated with other paternally-expressed loci regulated by the PWS-IC within the AS/PWS imprinted domain, we performed ChIP analysis of the mouse *Ndn* promoter for binding of these factors in Tg^ASdel^ or Tg^PWSdel^ cells. Potential binding sites for NRF-1, YY1, and Sp1 are present in the *Ndn* 5′ flanking region as determined by analysis with the TRANSFAC database. As shown in Fig. 7C, NRF-1, YY1, and Sp1 are all preferentially associated with the paternal *Ndn* allele. No potential NRF-2 binding sites are present in the *Ndn* gene and no binding of NRF-2 was detected at *Ndn* by ChIP analysis on mouse brain cells (data not shown). Thus, both *Mkrn3* and Ndn – two genes regulated by the PWS-IC – are bound by NRF's and YY1, suggesting these factors could contribute to the coordinate expression and regulation of *Mkrn3* and *Ndn* on the paternal chromosome by the PWS-IC. However, similar ChIP analysis of the mouse *Magel2* promoter region demonstrated little, if any, association of NRF-1 or YY1 with the promoter region in mouse brain, spleen, and Tg^ASdel^ or Tg^PWSdel^ cells (data not shown). This is consistent with sequence analysis of the *Magel2* promoter which showed an absence of canonical NRF-1, NRF-2, or YY1 sites.

### Chromosome Conformation Capture (3C)

**Figure 8:**
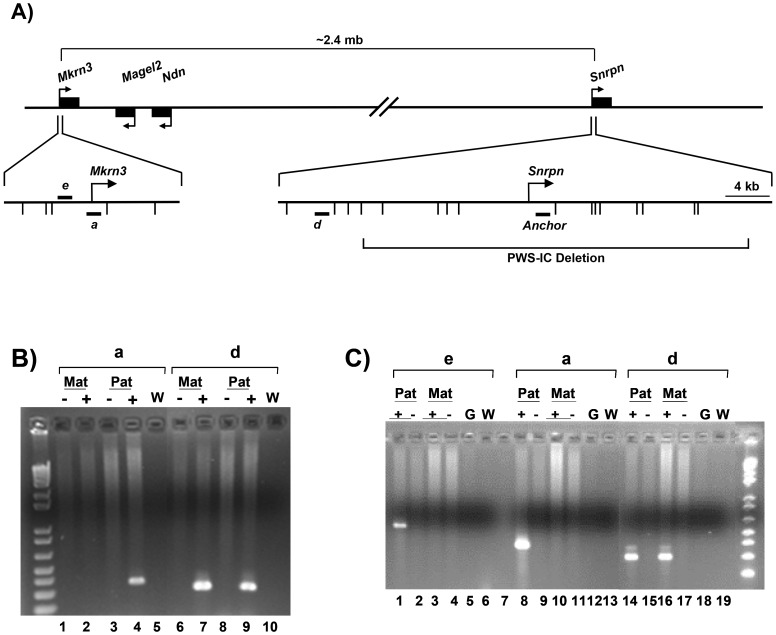
Chromosome conformation capture (3C) analysis of the *Mkrn3* locus. A) Diagram of 3C analysis of the *Mkrn3* locus showing the location of the anchor primer and primers *a*, *d*, and *e*. Bent arrows indicate transcription initiation sites. Short horizontal bars depict the location of primers *a*, *d*, *e*, and the anchor primer. Short vertical marks below the magnified *Mkrn3* and *Snrpn* 5′ regions indicate the location of EcoRI sites. The regions surrounding *Mkrn3* and the *Snrpn* transcription initiation site are shown approximately to scale. The long horizontal brackets show the approximate distance between the *Snrpn* and *Mkrn3* promoter regions, and the relative location of the 35 kb PWS-IC deletion in the *Snrpn* locus [18]. B) 3C analysis of Tg^PWSdel^ and Tg^ASdel^ fibroblasts using primers *a* and *d* with the anchor primer. The figure shows an ethidium bromide-stained agarose gel containing the products of PCR reactions between the anchor primer and the indicated primer. *Mat* indicates analysis of the maternal allele in Tg^PWSdel^ cells, *Pat* indicates analysis of the paternal allele in Tg^ASdel^ cells. “**−**” indicates a non-ligated control template, “**+**” indicates the ligated template, and “W” indicates a control PCR reaction with an equal volume of H_2_O substituted for a 3C template. C) 3C analysis of primary mouse brain cells. 3C analysis was performed on single-cell suspensions of newborn brains from mice carrying the 35 kb PWS-IC deletion on either the maternal or paternal chromosome. All designations are the same as those previously described. Primers *a*, *d* and the anchor primer were identical to those used in panel B. Primer *e* is located within the same EcoRI fragment as primer *a*. *Pat* denotes 3C templates from brain cells carrying the 35 kb PWS-IC deletion on the maternal chromosome, *Mat* denotes 3C templates containing the PWS-IC deletion on the paternal chromosome. “G” indicates control purified mouse genomic DNA.

The presence of NRF's and YY1 in both the PWS-IC and *Mkrn3* promoter region led us to examine whether or not these two loci separated by >2 mb of genomic DNA are in close physical proximity specifically on the paternal chromosome (Figure 8; [31]). 3C analysis was performed on Tg^PWSdel^ and Tg^ASdel^ fibroblasts to examine potential interactions between the PWS-IC and *Mkrn3* on the maternal and paternal chromosomes, respectively. 3C DNA templates were subjected to PCR using a primer from within the PWS-IC region (the “anchor” primer located 2.2 kb downstream from the *Snrpn* transcription initiation site) in conjunction with primers from the *Mkrn3* and *Snrpn* loci. PCR products were run on agarose gels and stained with ethidium bromide to visualize bands derived from amplification across novel junctions of ligated EcoRI fragments that were in close proximity in vivo. As shown in Fig. 8A, primers associated with the *Mkrn3* locus and upstream of the *Snrpn* promoter region were used in combination with the PWS-IC anchor primer. Primers *a* and *e* are located ∼1250 bp downstream and ∼3360 bp upstream of the *Mkrn3* transcription initiation site, respectively, and are contained on the same 5.2 kb genomic EcoRI fragment (which also contains the *Mkrn3* promoter). Primer *d* is located 17.9 kb upstream of the *Snrpn* promoter and ∼20 kb upstream of the anchor primer.

A readily detectable PCR product of the expected size was obtained using the anchor primer paired with primer *a* and the 3C template containing the paternal allele, demonstrating that a long-range interaction is occurring between the PWS-IC and the *Mkrn3* locus on the paternal chromosome (see lane 4 in Fig. 8B). No bands were detected with primer *a* using a 3C template containing only the maternal allele (lane 2). In addition, we detected a PCR product between the anchor primer and primer *d* on both the maternal and paternal chromosomes (see lanes 7 and 9, Fig. 8B), suggesting that the PWS-IC is either interacting non-specifically with this region of the *Snrpn* locus on both chromosomes because of proximity of this region to the PWS-C (a distance of ∼20 kb), or that on both the maternal and paternal chromosomes, the region containing primer *d* is engaging in a specific interactions with the PWS-IC and reflecting a previously unknown functional interaction; at this time, we cannot distinguish between these two possibilities. The result observed with primer *d* is also notable because it demonstrates that the lack of PCR products from the Tg^PWSdel^ (maternal) 3C template using primer *a* was not simply due to a general inability to PCR-amplify 3C templates generated from the Tg^PWSdel^ cells. All negative control lanes (including non-ligated template DNA preparations, and water controls containing no template DNA) showed no detectable PCR product, as expected. All PCR products from 3C templates were extracted from gels and cloned, then multiple clones from each PCR reaction were sequenced to verify that the expected novel ligation products between two distant genomic EcoRI fragments were amplified from the 3C templates. All of the sequenced clones for each PCR product (i.e., Fig 8B, lanes 4, 7, and 9) were the expected novel ligation products. Thus, the region containing the mouse *Mkrn3* locus is engaged in detectable long-range interactions with the PWS-IC region in vivo specifically on the paternal chromosome in immortalized mouse fibroblasts.

To determine whether or not this parent-of-origin interaction between *Mkrn3* and the PWS-IC also occurred in a normal mouse tissue, we performed a similar 3C analysis on primary newborn mouse brain cells. To distinguish between long-range interactions on the maternal chromosome from those on the paternal chromosome, we used brain cells from mice that carry the 35 kb PWS-IC deletion of the mouse *Snrpn* gene on either the paternal [18] or maternal chromosome [8]. The sequence for the 3C anchor primer is contained within this 35 kb PWS-IC deletion. Single-cell suspensions from dissected newborn brains were subjected to 3C analysis to detect interaction between the PWS-IC and *Mkrn3*. PCR primers *a*, *d*, and the anchor primer were identical to those used for 3C analysis of cultured fibroblasts (Figs. 8A and B); another *Mkrn3*-specific primer, primer *e*, located within the same EcoRI fragment as primer *a*, was also used in 3C analysis of brain cells. As shown in Fig. 8C, primers *a* and *e* in conjunction with the anchor primer yielded readily detectable PCR products exclusively from 3C templates containing only the paternal allele (lanes 1 and 8). These data suggest a long-range interaction in vivo between the *Mkrn3* locus and PWS-IC exclusively on the paternal chromosome in mouse brain cells, results similar to that in mouse fibroblasts. Again, primer *d* in conjunction with the anchor primer generated a readily detectable PCR product from both the maternal and paternal chromosomes of brain cells (Fig. 8C, lanes 14 and 16), as it did in fibroblasts. Cloning and sequencing the 3C PCR products from brain cells showed that 75–100% of the cloned products from a given PCR reaction were the expected novel ligation products between the EcoRI fragment containing the anchor primer and the EcoRI fragments containing primers *a*, d, or e (Fig. 8C, lanes 1, 8, 14, and 16). Rabinovitz et al. have recently shown a similar long-range interaction between the PWS-IC and *MKRN3* locus on the paternal chromosome in the human AS/PWS domain of lymphoblasts [46]. Taken together, these studies suggest that the mechanism of imprinting of the *Mkrn3* locus, and possibly other distal paternally expressed genes in the AS/PWS domain, involves differential spatial organization and interactions of the PWS-IC on the maternal and paternal chromosomes.

## Discussion

### Differential chromatin structure

Previous studies of the PW-IC region in human lymphoblasts have shown it to be associated with two DH sites exclusively on the paternal chromosome, one located in the *SNRPN* promoter region, and the other within the 1^st^ intron of *SNRPN* just downstream of the PWS-SRO and termed the CAS [17]. The location of these DH sites on the paternal allele suggested a possible role for these sites in human PWS-IC function, and we have proposed that the PWS-IC on the paternal chromosome consists of multiple DH sites that act as positive regulators of genes expressed from the paternal chromosome [17]. The murine PWS-IC has recently been localized to a 6 kb interval spanning the 5′ flanking region, promoter, and 5′ portion of the 1st intron of *Snprn* [19]. Therefore, we examined DH sites in chromatin within and flanking this interval on the maternal and paternal chromosomes in primary brain cells to identify potential functional components of the PWS-IC. We identified a pattern of six major allele-specific DNase I hypersensitive sites in the mouse *Snrpn* locus, all of which are on the paternal allele, and five of which (DH sites 1–5) are located within the 6 kb interval that currently defines the murine PWS-IC. Thus, we hypothesize that DHS 1–5 are major components of the PWS-IC and play a role in mediating PWS-IC function. DHS2 and DHS5 in mouse brain cells correspond to the two DH sites found in human lymphoblasts (the promoter region and CAS, respectively). Comparing our data with *Snrpn* DNase I hypersensitivity data from the UCSC Genome Browser (http://genome.ucsc.edu), strong distinct peaks of hypersensitivity corresponding to DH sites 2–5 are reported in the Genome Browser in 8 week adult mouse brains, day 14.5 embryo brains, and mouse ES cells, and less distinct peaks at these DH sites in 8 week adult mouse cerebrum and cerebellum tissue. The parent-of-origin of the DH sites in the UCSC Genome Browser was not determined. Weak or no peaks of hypersensitivity are reported in the Genome Browser for DH sites 1 and 6 in most tissue and cell samples analyzed. However, analysis of ES cells shows two strong peaks of hypersensitivity upstream of the *Snrpn* promoter at positions −1.3 kb and −3 kb. The peak at −3 kb corresponds to DH site 1 in our newborn brain cells, but we have not detected a hypersensitive site at position −1.3 kb in our assays of newborn brain on either the paternal or maternal alleles. Furthermore, other brain samples reported on the Genome Browser also do not detect this −1.3 kb DH site. Thus, an apparent ES cell-specific DH site of unknown allele-specificity is located between DHS1 and DHS2. In addition, no other cell or tissue shown on the Genome Browser show a strong DH site corresponding to DHS6 in our primary brain cells, though multiple cells/tissues show a weak signal corresponding to the position of DHS6 from our study. We cannot explain why DHS1 is readily detected only in newborn brain and ES cells, or why no other reported cell or tissue other than newborn brain cells shows a major hypersensitive site corresponding to DHS6. Based on our finding of six DH sites in the PWS-IC of primary mouse brain cells, we would predict that the PWS-IC/*SNRPN* gene in human primary brain cells contains more than the two DH sites we identified in on the paternal chromosome in human lymphoblasts [17].

The location of the six major DH sites we have identified here may provide insight into the effects of various targeted deletions of the PWS-IC in mice (see Fig. 1) assuming the DH sites identified here are candidates for the functional components of the PWS-IC. The 6 kb interval of the mouse *Snprn* locus that defines the minimal PWS-IC in the mouse contains DH sites 1 through 5 and yielded a complete imprinting defect in the AS/PWS domain when paternally transmitted [19]. This indicates that one or more cis-acting elements essential for normal PWS-IC function are located within this 6 kb deleted region, most likely within one or more of these five DH sites. A targeted deletion containing 0.9 kb of the *Snrpn* promoter region and exon 1, and including only DHS2, showed no disruption of either imprinted gene expression nor DNA methylation patterns when paternally (or maternally) inherited [38], suggesting that the *Snrpn* promoter region (DHS2) is not essential for PWS-IC function in the mouse. However, a 4.8 kb deletion of the *Snrpn* 5′ region that included the same 0.9 kb region of the promoter as well as sequences ∼1.4 kb upstream and ∼2.5 kb downstream of the promoter yielded mice exhibiting growth retardation, a partial imprinting defect, and 40–50% perinatal lethality when paternally inherited [38]. This 4.8 kb deletion removed DH sites 2–5 (see Fig. 1D) as well as the DH site at position −1.3 kb reported for ES cells in the UCSC Genome Browser. This would suggest that sequences within this 4.8 kb deletion contain elements that contribute significantly to PWS-IC function in the mouse, and that DH sites 2–5 could contain such elements. Furthermore, this deletion indicates that other sequences within the minimal 6 kb PWS-IC can provide partial PWS-IC function in the absence of this 4.8 kb region that includes DH sites 2–5. The candidates for these other sequences are DH sites 1 and/or 6, and indicate that full PWS-IC function is likely to be dependent upon cis-acting components in multiple DH sites. A fourth targeted deletion in the *Snrpn* locus which extended from *Snrpn* intron 1 downstream to *Ube3A* and removed DH site 6 showed no apparent effect on imprinting [39]. This demonstrates DH site 6 is not essential for PWS-IC function in the presence of DH sites 1–5 and that in the absence of DH site 6 the remaining DH sites can provide full PWS-IC function. This would then suggest that DHS1 is providing the partial imprinting function observed in the 4.8 kb deletion.

### Trans-acting factors associated with the paternal PWS-IC

The PWS-IC has been postulated to act as a positive regulator of paternally-expressed genes within the AS/PWS domain [10]. This activity is likely to be mediated by regulatory factors associated with the 6 kb interval that defines the minimal murine PWS-IC. We now have identified transcription factors bound within the mouse PWS-IC, as well as the promoters of *Mkrn3* and *Ndn*, two distal genes regulated by the murine PWS-IC located >2 mb from the IC. All factors are bound exclusively on the paternally-inherited alleles. Strikingly, all of these regulatory regions are associated with nuclear respiratory factors, either NRF-1 or NRF-2. NRF-1 is associated with the *Snrpn* promoter region, the CAS (the conserved enhancer sequence located within the 1^st^ intron of *SNRPN*/*Snprn*; [17]), and the *Ndn* promoter, while the *Mkrn3* promoter is bound by NRF-2 rather than NRF-1. Though unrelated in composition and DNA sequence recognition, NRF-1 and NRF-2 play a similar role in activation of nuclear genes involved in mitochondrial biogenesis and function [42], [47], [48], [49], [50]. NRF-1 is a 68 kD polypeptide that as a homodimer recognizes the binding sequence YGCGCAYGCGCU [47]. NRF-2, also known as GABP in the mouse [51], [52], is a multimeric DNA-binding factor that binds to the sequence CGGAAG [47], with the core binding sequence of GGAA typical of the ETS-domain transcription factor family. Genes associated with mitochondrial function and regulated by nuclear respiratory factors can be bound and activated by NRF-1 (e.g., human cytochrome c), by NRF-2 (e.g., mouse cytochrome oxidase subunit IV), or both (e.g., human succinate dehydrogenase subunit B). Genes other than those involved in mitochondrial biogenesis and respiration have also been shown to be regulated by NRF-1 and NRF-2, such as the human FMR1 gene associated with fragile X syndrome [53]; reported functions of NRF-2/GABP include regulating B lymphocyte and myeloid development [52], [54], cell cycle progression [55], and formation of neuromuscular junctions [56]. Overall, our analysis has shown that multiple regulatory regions within the imprinted AS/PWS domain are bound by nuclear respiratory factors and/or YY1. The finding that YY1 is a commonly bound transcription factor in multiple regulatory regions across the AS/PWS domain, including the PWS-IC region, is intriguing because clustering of YY1 binding sites has been reported to occur in a variety of imprinting control regions [43], [44]. Differentially methylated regions (DMR's) of several epigenetically regulated loci (including *Peg3*, *Nespas*, *Xist*, and *Tsix*) have been shown to contain clustered YY1 binding sites [43], and YY1 knockdown studies in cultured cells showed altered gene expression in the imprinted *Peg3* and *Gnas* domains, as well as the *Snrpn* locus [44]. Thus, YY1 may have a significant role in the regulation of a subset of imprinted domains [57]. Identification of functional YY1 binding sites in the PWS-IC region is also intriguing because YY1 has recently been reported to anchor Xist RNA to the inactive X chromosome [58], suggesting a role for YY1 in the localization of non-coding regulatory RNA's.

We previously identified the intronic CAS in the human *SNRPN* gene as a conserved cis-acting regulatory region associated with DNase hypersensitivity and enhancer activity; however, it was located just downstream and outside of the human PWS-SRO [17]. The recent delineation of the minimal murine PWS-IC to a 6 kb interval in the *Snprn* locus [19] now shows the murine CAS to be contained within this interval. This provides evidence that the CAS is likely to be a functional component of the human PWS-IC, and that the contribution of the CAS to PWS-IC function may be mediated, at least in part, by NRF-1 and YY1. The ChIP-positive YY1 binding site associated with DHS6 (see Fig. 3) is located ∼1.8 kb downstream and outside of the minimal 6 kb PWS-IC interval, and its deletion has no effect on imprinting [39]. Thus, its role and contribution to PWS-IC function is unclear.

In vivo footprinted sites in *Snrpn* identified here are associated with several cis-acting elements identified in earlier published reports that used the minimal *Snrpn* promoter in either transfection studies or in a mini-transgene [40], [59], [60]. Though these reports did not identify specific transcription factors associated with these regulatory sequences, some of these cis-acting elements correlate with regulatory sites and regions detected in the current study. A 7 bp element (SBE; gcgcatg) that is absolutely required for *Snrpn* promoter activity [40], [59] coincides with footprint P3 and is associated with an NRF-1 binding site (Fig. 2). A sequence identified as being one of two de novo methylation signals (DNS1; agggagc) in a *Snrpn* mini-transgene [60] is located adjacent to footprint P4 (Fig. 2). P4 is associated with overlapping NRF-1 and Sp1 binding sites and also lies adjacent to a sequence conserved between the human and mouse promoters that is footprinted in the human *SNRPN* promoter [17]. Other *cis*-acting elements (ADS, DNS2) identified in these mini-transgene studies were not footprinted in our experiments; at this time we cannot determine if these latter cis-acting sites are simply not occupied by regulatory proteins in the cells we analyzed, or if trans-acting factors associated with these sites do not interact directly with guanine residues and, therefore, were not detected by DMS footprinting. Analysis of imprinting of the *Snrpn* mini-transgene also led to the identification of sequences in the 1^st^ intron of *Snrpn* that are identical to the cis-acting elements in the *Snrpn* promoter that were required for correct imprinting of the mini-transgene [60]. The location of at least one of these intronic elements (DNS) is in the vicinity of DHS3.

The presence of both NRF-1 and NRF-2 in multiple regulatory regions and multiple genes within the AS/PWS domain suggests a functional relationship between regulation of the AS/PWS domain and regulation of cellular respiration and/or mitochondrial biosynthesis. This potential relationship between energy metabolism and AS/PWS is intriguing because of the obesity phenotype characteristic of PWS patients as well as the late onset obesity seen in Tg^ASdel^ mice which carry a deletion of the murine AS/PWS region on the maternally-inherited chromosome [20], [45]. Furthermore, a report suggests that changes in expression of many genes, particularly those related to energy metabolism, may contribute to the failure to thrive seen in infants with PWS [61]. However, it is not immediately apparent how NRF-mediated co-regulation of respiratory genes and paternal genes in the AS/PWS domain may be associated with the PWS and Tg^ASdel^ phenotypes, or how mutation or disruption of imprinting in the AS/PWS domain could affect regulation of energy metabolism.

It appears that an exception to regulation of paternally-expressed genes by nuclear respiratory factors and YY1 may be the mouse *Magel2* locus for which we find no strong evidence for binding of these factors to the promoter region on the paternal chromosome in mouse brain, spleen, Tg^ASdel^ or Tg^PWSdel^ cells. It is possible that regulation of *Magel2* may include additional unidentified regulatory region(s) outside of the promoter region that bind YY1 and/or NRF's, or that *Magel2* is regulated by the PWS-IC via a different mechanism than *Mkrn3/Ndn* that does not involve YY1 and NRF's.

In addition to ubiquitous transcription factors (e.g., NRF-1, NRF-2, YY1, Sp1), it is likely that tissue/cell type-specific transcription factors also interact with the PWS-IC and/or the regulatory regions of its target genes (e.g., *Mkrn3*, *Ndn*) on the paternal chromosome because of the tissue-specific pattern of expression in brain cells exhibited by these target genes [22], [34], [35], [36], [37]. However, our in vivo footprinting of the *Snrpn* promoter region in brain cells did not identify binding sites of known neuronal transcription factors.

### Spatial organization of the AS/PWS domain

An emerging theme in the coordinate regulation of multi-gene domains – including imprinted genes [62], [63] – is the role of spatial organization and nuclear localization [64],. We previously speculated [17] that multiple DH sites associated with the PWS-IC on the paternal chromosome may function by forming a chromatin holocomplex in conjunction with regulatory regions of its target genes (e.g., *Mkrn3*, *Ndn*) into a structure similar to an active chromatin hub (ACH; [65], [66], [67], [68]). The long-range interactions between the PWS-IC and its target genes in forming such a holocomplex would facilitate establishment of the paternal epigenotype and coordinate expression of these paternal genes. Moreover, formation of this holocomplex would result in the localization of the PWS-IC and its target genes on the paternal chromosome to a transcription factory to coordinately activate transcription of these genes exclusively on the paternal chromosome [69], [70]. This mechanism for PWS-IC function would be analogous to that of the β-globin locus control region (LCR) which engages in long-range interactions with β-globin genes and is required for localization of the β-globin locus to transcription factories during the course of erythroid development [71]. Taken together, the results presented here for the murine PWS-IC, as well as 3C studies reported for the human PWS-IC [46], are all consistent with such a scenario for PWS-IC function. The DH sites in the PWS-IC facilitate long-range interactions of the PWS-IC with regulatory regions of distal target genes on the paternal chromosome to form an ACH and localize this holocomplex to a transcription factory, thereby establishing a transcriptionally active state of these paternal genes. Our finding of multiple regulatory regions across the AS/PWS domain commonly bound in a parent-of-origin fashion by NRF's and/or YY1 further supports a transcription factory-based model for PWS-IC function whereby the PWS-IC co-localizes with paternally-expressed genes to a transcription factory(s) enriched in NRF's and YY1 (as well as other positive regulators of transcription such as general transcription factors, histone acetyltransferases, Sp1, RNA pol II, etc.). This would be consistent with the proposed role of the PWS-IC functioning as a positive regulator of paternally-expressed AS/PWS genes [10]. In contrast, formation of an ACH-like holocomplex with the PWS-IC on the maternal chromosome and localization to a transcription factory would not occur, presumably due to the influence of the AS-IC, leading to establishment of the maternal epigenotype. Though we did not detect NRF and YY1 binding to the *Magel2* promoter region, this does not preclude the PWS-IC-mediated localization of *Magel2* to a NRF/YY1-containing transcription factory by a mechanism not involving these factors.

Co-localization to neuronal transcription factories enriched in NRF-1 and NRF-2 has been proposed as a mechanism for coordinately regulating all ten nuclear genes encoding subunits of cytochrome c oxidase which are regulated by NRF's [72]. Thus, it is also conceivable that in neuronal cells at least a subset of paternally-expressed genes in the AS/PWS domain may co-localize to (and be co-regulated by) a transcription factory that is also associated with genes involved in mitochondrial function and respiration that are regulated by NRF's.

Rabinovitz et al. used 3C analysis of the human AS/PWS domain to demonstrate an allele-specific long-range interaction between the PWS-IC and a region located between the *MKRN3* and *MAGEL2* genes [46]. They proposed a model in which the PWS-IC physically interacts with this region specifically on the paternal chromosome, with looping out of the intervening DNA. Based upon DNA methylation analysis of the interacting region between *MKRN3* and *MAGEL2*, they further suggested this interaction may be regulated by the methylation state of the PWS-IC rather than the methylation state of the region interacting with the PWS-IC. They did not report whether or not they assayed PWS-IC interactions with the *MKRN3* promoter region or with each of the other upstream paternally-expressed genes. Our 3C analyses indicated a similar paternal-specific interaction of the murine PWS-IC with the *Mkrn3* locus, which in the human AS/PWS domain is ∼20 kb further from the PWS-IC than the interacting site identified by Rabinovitz et al. It is conceivable that the difference between our results and those of Rabinovitz et al. is simply a species-specific difference in the manner by which the PWS-IC interacts with the paternally-expressed distal gene cluster on the paternal chromosome. Alternatively, the results from both 3C studies together may reflect: 1) an essentially equivalent result because of the low resolution of 3C assays, suggesting that a broad region containing and surrounding *MKRN3*/*Mkrn3* lies in proximity to the PWS-IC on the paternal chromosome; 2) a three-dimensional organization on the paternal chromosome in which multiple regions (presumably regulatory regions) across the distal paternally-expressed gene cluster all lie in proximity to the PWS-IC (perhaps within the context of a transcription factory); and/or, 3) the possibility that interactions with the PWS-IC on the paternal chromosome are dynamic and that at different times within a given cell different regions within the distal gene cluster transiently interact with the PWS-IC (within a transcription factory). The fact that the region of the mouse *Mkrn3* locus that interacts with the PWS-IC contains a DMR, while Rabinovitz et al. find the interacting region downstream of the human *MKRN3* gene to be unmethylated on both alleles, further suggest that methylation patterns of the region interacting with the PWS-IC are unlikely to govern this interaction.

Currently, a direct role for transcription factors in PWS-IC function has not yet been established. However, coordinate and developmental regulation of other multigene domains such as the β-globin domain and T helper type 2 cytokine locus have been shown to be dependent upon specific transcription factors that also mediate long-range intrachromosomal interactions within these domains [32], [73], [74], [75]. Given our findings that YY1 and NRF's are bound at multiple regulatory sites across the AS/PWS domain on the paternal chromosome including the PWS-IC, we propose that these trans-acting factors play a significant role in PWS-IC function. We further speculate that other as yet unidentified factors associated with the DH sites of the PWS-IC may also contribute to PWS-IC activity.
